# Supplementary material for: Structural characteristics of alpha-fetoprotein, including N-glycosylation, metal ion and fatty acid binding sites
Source: Commun Biol. 2024 Apr 27;7:505. doi: 10.1038/s42003-024-06219-0 (PMC11055904; doi:10.1038/s42003-024-06219-0)
Supplement: Supplementary file 2 — Supplementary Information [file 42003_2024_6219_MOESM2_ESM.pdf]

Supplementary Information

Structural characteristics of alpha-fetoprotein, including N-glycosylation, metal ion and fatty acid binding sites

Kun Liu, Cang Wu, Mingyue Zhu, Junnv Xu, Bo Lin, Haifeng Lin,

Zhongmin Liu and Mengsen Li

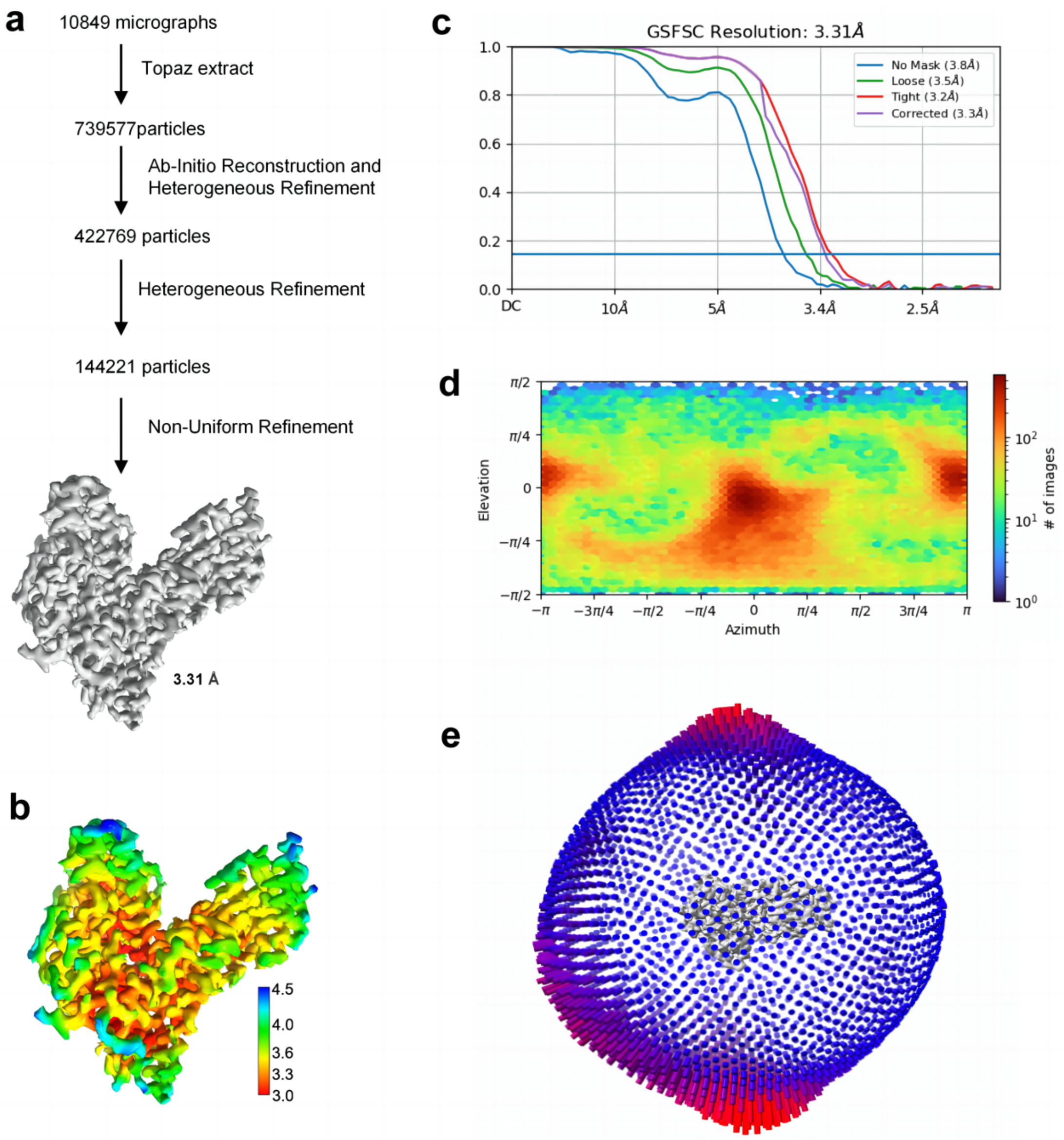

Supplementary Fig. 1 Cryo-EM analysis of hAFP. a Processing workflows of cryo-EM data. **b** Local resolution estimation of the final 3D density map of hAFP. **c** Gold-standard Fourier Shell correlation (FSC=0.143) curve after 3D refinement. d Particle orientation distributions in the last iteration of the structural refinement. e Euler angle distribution of the reconstruction.

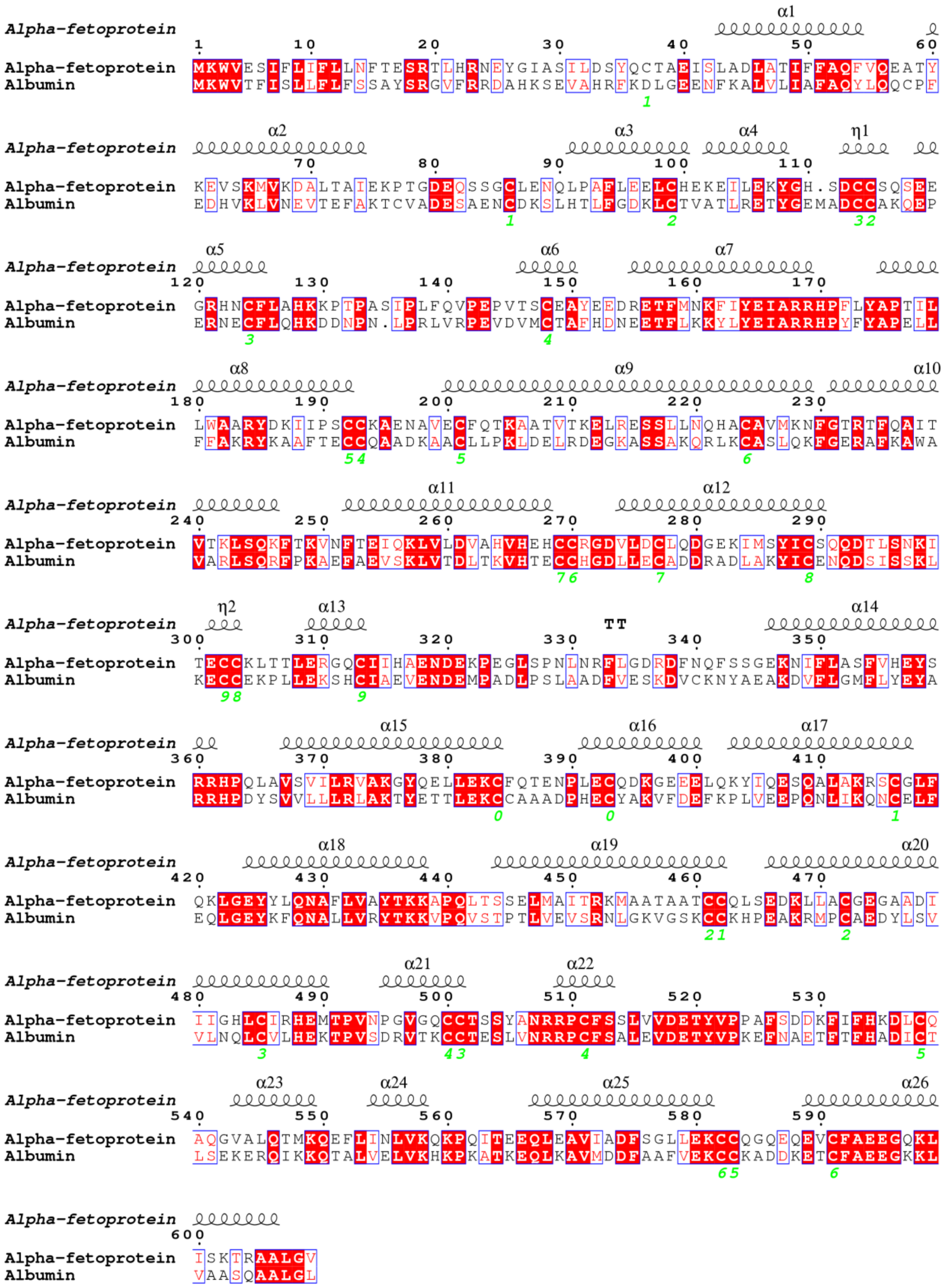

Supplementary Fig. 2 Sequence alignment of AFP and HSA. Representative secondary structural elements of AFP are displayed above the sequences. Invariant and highly conserved residues are shaded red and colored red, respectively. Cystine residues involved in disulfide bond formation are represented by the same green-colored numbers.

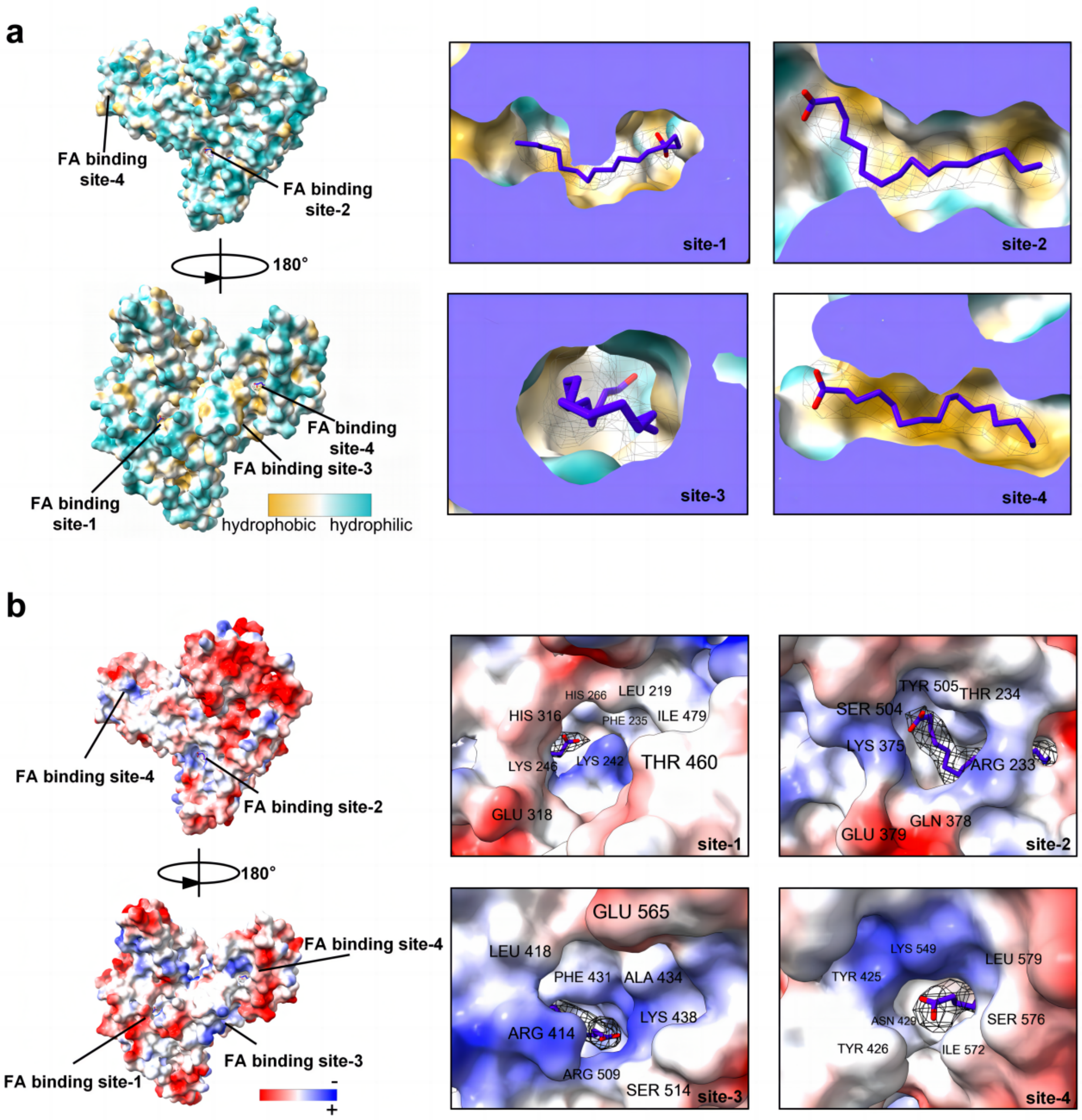

Supplementary Fig. 3 Analysis of AFP's hydrophobicity and electrostatic potential surface. a The hydrophobicity of fatty acid binding pockets in AFP. **b** AFP is displayed in surface plot and is coloured according to its coulombic potential. The amino acids located in fatty acid binding pockets entry is marked.

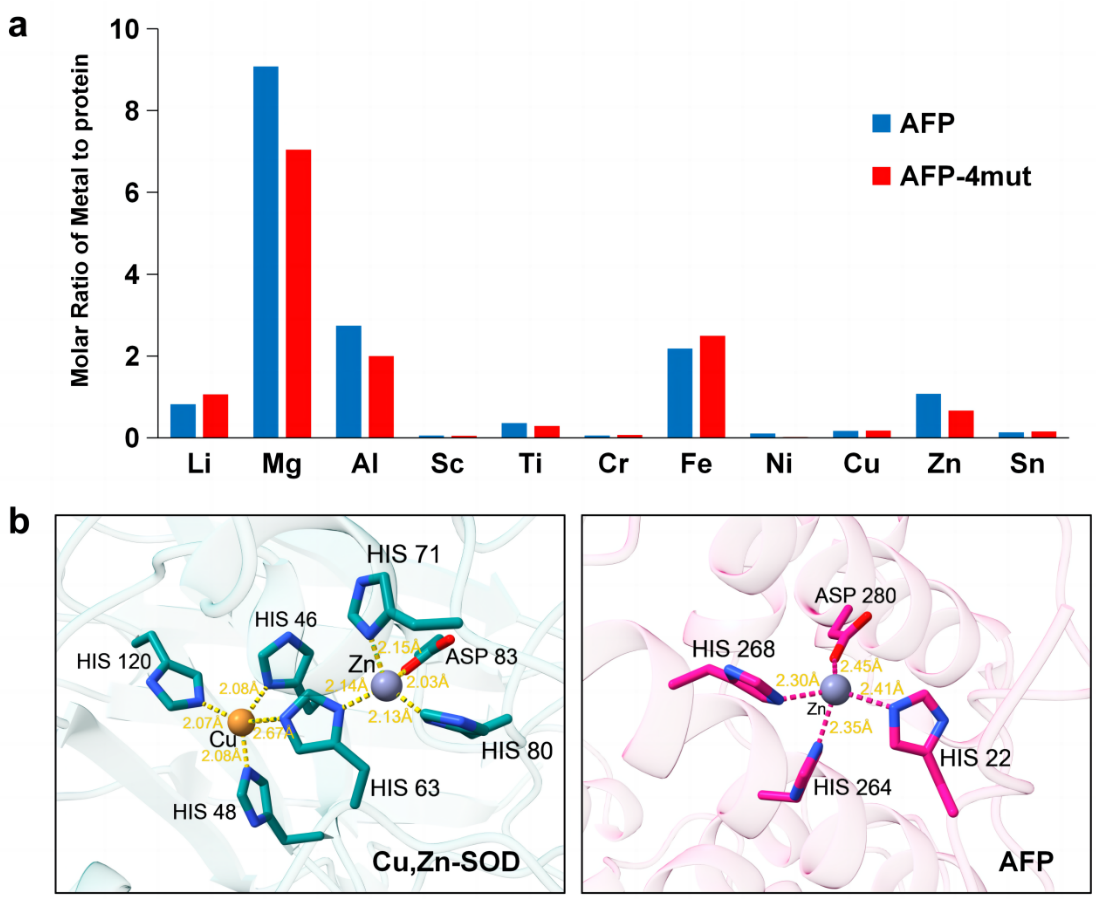

c CheckMyMetal(CMM) report for PDB code: vC52pu_3616

ID	Res.	Metal	*Occupancy*	*B factor (env.)*[1]	*Ligands*	*Valence*[2]	*nVECSUM*[3]	*Geometry*[1,4]	*gRMSD(°)*[1]	*Vacancy*[1]	Bidentate	Proposed Metals
A:701	ZN	Zn	1.0	71.1 (43.5)	O_1N_3	0.7	0.33	Tetrahedral	20.2°	0	0.0	Cu(6), Na(5), K(4), Mn(4), Fe(4), **Zn(4)**, Co(3), Mg(2), Ca(2), Ni(2)
		**Legend:**	Not applicable	**Outlier**	*Borderline*	Acceptable						

Supplementary Fig. 4 Content detection and structure verification of metal ions. **a** Metal content in AFP was identified by ICP-MS. The horizontal axis of the histogram represents the metal elements. Some elements with lower content or additional additions are not shown. The vertical axis represents the molar ratio of metal to protein. AFP-4mut indicates four mutations: H22A, H264A, H268A, and D280A. The decrease in the molar ratio of AFP-4mut to metal indicates that the metal is binding to AFP. **b** Atomic models depicting the binding sites of metal ions in Cu,Zn-SOD (5K02,dark cyan) and AFP (8X1N, pink). The yellow dashed lines represent the distance of the ion to the residue. **c** Validation of AFP-Zn^{2+} by the CheckMyMetal server. The website is https://cmm.minorlab.org/, PDB code: 8X1N.

Supplementary Table 1. Analysis of AFP glycosylation sites and corresponding sugar types.

Protein	Type (Position)	Peptide	Glycans NHFAGNa	Modification Type(s)	Score	Intensity	Percentage (%)
AFP(293F)	N-Glyco (251)	TKVN*FTEIQKL	HexNAc(1)Fuc(1)	N[+349]	649.57	4.63E+09	10.60
			HexNAc(1)	N[+203]	440.17	1.76E+09	4.03
			HexNAc(4)Hex(5)	N[+1623]	395.34	2.13E+09	4.87
			HexNAc(3)Hex(4)Fuc(1)	N[+1404]	385.45	6.33E+08	1.45
			HexNAc(3)Hex(3)Fuc(1)	N[+1241]	369.47	2.61E+08	0.60
			HexNAc(4)Hex(5)Fuc(1)	N[+1769]	367.92	6.48E+09	14.83
			HexNAc(5)Hex(4)Fuc(1)	N[+1810]	360.88	1.17E+10	26.82
			HexNAc(5)Hex(4)Fuc(2)	N[+1956]	322.91	4.50E+08	1.03
			HexNAc(2)Hex(7)	N[+1541]	310.72	3.26E+08	0.75
			HexNAc(2)Hex(6)	N[+1378]	310.32	2.39E+08	0.55
			HexNAc(2)Fuc(1)	N[+552]	308.84	8.23E+07	0.19
			HexNAc(5)Hex(4)	N[+1664]	298.46	5.33E+09	12.19
			HexNAc(5)Hex(5)Fuc(1)	N[+1972]	279.22	2.21E+09	5.06
			HexNAc(4)Hex(4)Fuc(1)	N[+1607]	277.36	1.86E+09	4.26
			HexNAc(2)Hex(8)	N[+1703]	259.33	1.41E+08	0.32
			HexNAc(5)Hex(3)	N[+1502]	258.68	1.37E+08	0.31
			HexNAc(4)Hex(3)Fuc(1)	N[+1445]	251.07	3.79E+09	8.68
			HexNAc(4)Hex(5)Fuc(2)	N[+1915]	221.96	1.39E+08	0.32
			HexNAc(5)Hex(3)Fuc(1)	N[+1648]	189.96	9.48E+08	2.17
			HexNAc(4)Hex(4)NeuGc(1)	N[+1768]	168.14	1.82E+08	0.42
			HexNAc(2)Hex(5)	N[+1216]	164.19	2.44E+08	0.56
AFP(Bel7402)	N-Glyco (251)	TKVN*FTEIQKL	HexNAc(1)Fuc(1)	N[+349]	660.47	2.83E+09	12.42
			HexNAc(2)Hex(7)	N[+1541]	314.34	1.33E+08	0.58
			HexNAc(4)Hex(5)	N[+1623]	301.37	4.04E+09	17.72
			HexNAc(4)Hex(5)Fuc(1)	N[+1769]	300.37	1.01E+10	44.44
			HexNAc(4)Hex(5)Fuc(2)	N[+1915]	282.61	2.64E+09	11.58
			HexNAc(4)Hex(4)	N[+1461]	278.80	2.30E+08	1.01
			HexNAc(2)Hex(8)	N[+1703]	270.40	1.83E+08	0.80
			HexNAc(4)Hex(4)Fuc(1)	N[+1607]	264.30	4.31E+08	1.89
			HexNAc(5)Hex(5)Fuc(1)	N[+1972]	215.88	1.12E+09	4.92
			HexNAc(5)Hex(4)Fuc(1)	N[+1810]	199.67	7.03E+08	3.08
			HexNAc(5)Hex(4)Fuc(2)	N[+1956]	184.36	1.15E+08	0.50
			HexNAc(2)Fuc(1)	N[+552]	183.70	6.10E+07	0.27
			HexNAc(3)Hex(5)NeuGc(1)	N[+1727]	162.66	1.78E+08	0.78

Supplementary Table 2. Data processing details, refinement, and validation statistics.

Name	Sequence (5’-3’)
AFP-F	ATGAAGTGGGTGGAATCAATTT
AFP-R	AACTCCCAAAGCAGCACG
AFP-TY-F	TTCGGATCCGCCACCATGAAGTGGGTGGAATCAATTT
AFP-TY-R	ATGATGGTGGTGATGAACTCCCAAAGCAGCACG
Vector-F	CATCACCACCATCATCACCATCAC
Vector-R	GGTGGCGGATCCGAATTCCTTA
AFP(N251S)-F	AAGTTTACCAAAGTTTCTTTTACTGAAAT
AFP(N251S)-R	AACTTTGGTAAACTTCTGACTCAGT
AFP(H22A)-F	CTGAATCCAGAACACTGGCTAGAAATGAA
AFP(H22A)-R	CAGTGTTCTGGATTCAGTAAAATTTAGT
AFP(H264A)-F	GTCCTGGATGTGGCCGCTGTACATGAG
AFP(H264A)-R	GGCCACATCCAGGACTAGTTTCTGGAT
AFP(H268A)-F	GCCCATGTACATGAGGCCTGTTGCAGA
AFP(H268A)-R	CTCATGTACATGGGCCACATCCAGG
AFP(D280A)-F	TGGATTGTCTGCAGGCTGGGGAAAAA
AFP(D280A)-R	CCTGCAGACAATCCAGCACATCTCC

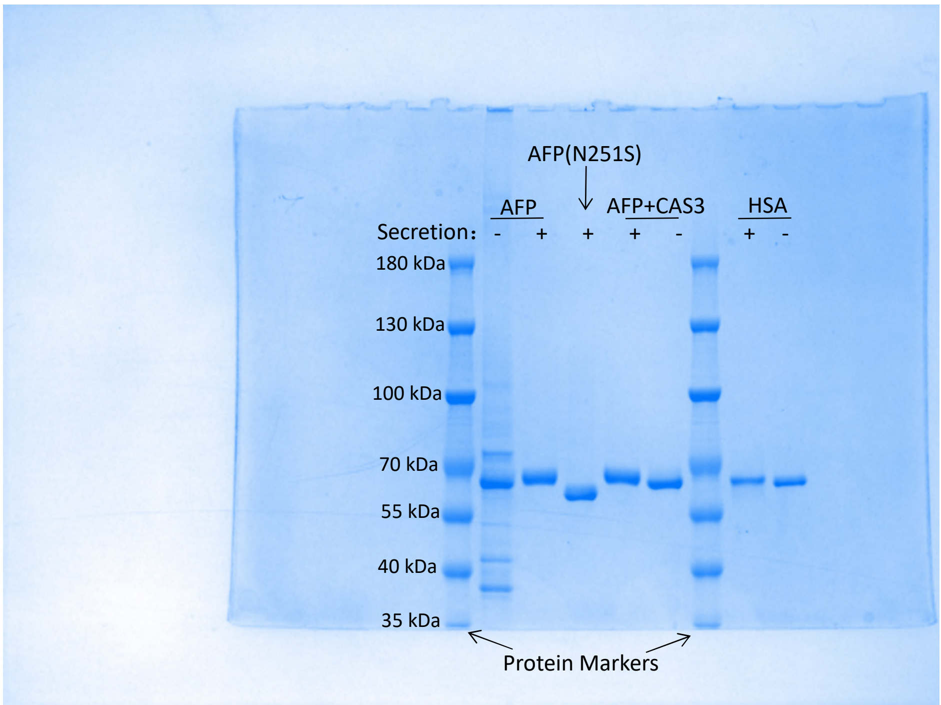

Supplementary Fig. 5 Uncropped gel (Fig. 3b)
